# Cardiac Microvascular Dysfunction in Women Living With HIV Is Associated With Cytomegalovirus Immunoglobulin G

**DOI:** 10.1093/ofid/ofy205

**Published:** 2018-09-11

**Authors:** Andreas Knudsen, Kristina Thorsteinsson, Thomas E Christensen, Philip Hasbak, Rasmus Sejersten Ripa, Inge Panum, Anne-Mette Lebech, Andreas Kjaer

**Affiliations:** 1 Department of Infectious Diseases, Copenhagen University Hospital, Hvidovre, Denmark; 2 Department of Clinical Physiology, Nuclear Medicine & PET, Copenhagen University Hospital, Rigshospitalet, Copenhagen, Denmark; 3 Cluster for Molecular Imaging, Department of Biomedical Sciences, University of Copenhagen, Copenhagen, Denmark; 4 Department of Clinical Microbiology, Copenhagen University Hospital, Hvidovre, Denmark; 5 Department of Infectious Diseases, Copenhagen University Hospital, Rigshospitalet, Copenhagen, Denmark

**Keywords:** cardiac microvascular, cytomegalovirus, HIV

## Abstract

**Background:**

People living with HIV (PLWH) appear to be at increased risk of cardiovascular disease (CVD), and this is possibly more pronounced in women living with HIV (WLWH). In the general population, men are more likely to develop obstructive coronary artery disease (CAD), and women often present with a nonobstructive pattern with cardiac microvascular dysfunction. We investigated cardiac microvascular function in men and women living with HIV and tested for association with cytomegalovirus (CMV) immunoglobulin G (IgG), as this has been associated with CVD in PLWH.

**Methods:**

In a cross-sectional study, 94 PLWH on antiretroviral therapy were scanned with ^82^Rb positron emission tomography/computed tomography at rest and during adenosine-induced stress, which enables the quantification of the myocardial flow reserve (MFR). CMV IgG was measured in plasma.

**Results:**

WLWH had significantly lower MFR compared with men living with HIV (MLWH; *P =* .003), and >45% of the women had an MFR indicative of cardiac microvascular dysfunction, whereas this was only true for 24% of men (*P =* .03). CMV IgG concentrations were inversely associated with MFR among WLWH but not MLWH (*P =* .05 for interaction).

**Conclusions:**

In this first study comparing MFR in women and men living with HIV, we found that WLWH had significantly lower MFR than MLWH and 45% of the women had cardiac microvascular dysfunction despite younger age and lower cardiovascular risk. Furthermore, CMV IgG was inversely associated with MFR among women but not men. This calls for attention to CVD among young WLWH even with low cardiovascular risk.

People living with HIV (PLWH) appear to be at increased risk of cardiovascular disease (CVD), with manifestations such as myocardial infarction (MI) [1] and heart failure [2]. Some studies suggest that this risk is even more pronounced in women living with HIV (WLWH) compared with men living with HIV (MLWH), but the mechanism behind this sex-based difference in risk is unclear [2–5]. The pathogenesis behind the increased risk of CVD associated with HIV infection is not fully understood but seems to be related to a higher prevalence of “traditional” cardiovascular risk factors, the antiretroviral therapy (ART) and immunologic changes involved in chronic infection with HIV [6]. Furthermore, cytomegalovirus (CMV) infection has been associated with risk of cardiovascular disease in the general population [7], and as co-infection with CMV is very common among PLWH [8], it has been speculated that CMV could contribute to CVD among PLWH. Indeed, both CMV-specific T-cell responses and CMV immunoglobulin G (IgG) have been associated with vascular changes among PLWH [9, 10].

Studies from the general population find that the pathogenesis and phenotype of CVD may differ between men and women [11], where men are more likely to develop flow-limiting atherosclerotic coronary artery disease but women are more likely to present with a nonobstructive pattern and a high degree of cardiac microvascular dysfunction (CMD) [11]. This may partly relate to anatomical differences in coronary diameter, higher coronary blood flow, and higher endothelial shear stress [12].

The gold standard for the measurement of the cardiac microvascular function and myocardial blood flow/perfusion is dynamic positron emission tomography/computed tomography (PET/CT) imaging, which enables the quantification of absolute myocardial perfusion in mL/g/min by intravenous injection of a perfusion positron–emitting tracer. Therefore, we conducted the first comparison of the cardiac microvascular function quantified as the myocardial flow reserve (MFR) by ^82^Rb PET between WLWH and MLWH. The MFR quantifies the vasodilator function of the cardiac circulation, and in the general population, a decreased MFR has shown to be associated with major adverse cardiac events (MACEs) among women even in the absence of obstructive coronary disease [13, 14]. In addition, we studied CMV IgG quantified in plasma to investigate the hypothesis that CMV IgG could influence cardiac microvascular function.

## METHODS

### Participants

MLWH were recruited from a previously described cohort [15], and perfusion data have been presented elsewhere [16]. WLWH were all recruited from the Study on HIV, cervical Abnormalities and infections in women in Denmark (SHADE), as described elsewhere [17]. Inclusion criteria were age ≥18 years and ART >12 months. Exclusion criteria were (i) asthma, (ii) pregnancy, or (iii) alcohol or drug abuse that could affect the ability to adhere to the protocol. Ninety-four PLWH comprising 50 men and 44 women underwent ^82^Rb PET/CT between August 2012 and February 2014.

### Ethics

The study was approved by the Scientific Ethics Committee of the Capital Region of Denmark (protocol number H-C-2008–060) and complied with the Declaration of Helsinki. All study participants received oral and written information and gave written consent before inclusion.

### PET Imaging

The detailed methodology of the ^82^Rb PET/CT myocardial perfusion imaging (MPI) has been described previously [16]. In brief, all study participants were asked to abstain from caffeine and theophylline-containing substances and medications for 12 hours before imaging, and abstinence was confirmed before the examination. Electrocardiography-gated MPI was performed during rest and stress conditions in a single session on a Siemens Biograph mCT/PET 128-slice scanner (Siemens Helthcare, Knoxville, TN). Study participants were stressed using adenosine for 6 minutes, and the stress ^82^Rb infusion was initiated 2.5 minutes after the start of the adenosine infusion. Low-dose CT for attenuation correction was performed before the rest study and after the stress study if required. Coronary artery calcium score (CACS) images were acquired as per clinical routine from a noncontrast breath-hold CT. The CACS was calculated according to the Agatston score using a threshold of 130 Hounsfield units (HU) [18]. Quantitative myocardial blood flow (MBF) was performed using *syngo* software (Siemens Healthcare, Knoxville, TN), which is based on a single-compartment model for ^82^Rb kinetics [19] to obtain the absolute MBF in milliliters per gram of tissue per minute. MFR was defined as MBF during maximal hyperemia (stress), obtained by the infusion of adenosine divided by MBF during rest. The MFR was corrected for baseline cardiac work by dividing the rest MBF by the rate pressure product (RPP), which is the systolic blood pressure times the heart rate, multiplied by 10 000 [20]. MFR was considered normal if ≥2.0, and values <2.0 reflected CMD for both men and women [13].

Semiquantitative analysis for detection of perfusion defects was computed as a summed stress score (SSS) according to the AHA 17 myocardial segment model [21] automatically with Corridor4DM (INVIA, Ann Arbor, MI). An SSS was considered abnormal if >4 [22]. Left ventricular ejection fraction (LVEF) was calculated automatically with Corridor4DM.

### Biomarkers and CVD Risk Score

CD4 cell counts, HIV RNA levels, creatinine, and serum lipids were determined routinely on blood and plasma when collected and information obtained from the study participants’ medical records. Information on comorbidities and medication was obtained by questionnaire and medical records. Framingham risk score (FRS) was calculated as the 10-year risk of coronary heart disease according to published definitions [23].

All study participants had plasma samples taken in a fasting state at the time of PET/CT and stored at –80°C until analysis of CMV IgG, which was performed by an automatic instrument (COBAS6000, Roche Diagnostics GmbH, and Mannheim, Germany) using an electro-chemiluminescence assay (cobas, Roche Diagnostics GmbH, Mannheim, Germany). The test results are quantitative and are provided in units/mL (U/mL).

The testing was performed according to the recommendations of the manufacturer.

### Statistics

Data are shown as mean ± standard error of the mean or median (range). Continuous variables were compared using an unpaired *t* test after log_10_-transformation to obtain a normal distribution. Categorical variables were compared by chi-square test. Associations were analyzed with both logistic (categorical variables) and multiple linear regression (continuous variables after log_10_-transformation) models. In the multiple regression analysis, adjustments were made for factors known to be involved in CVD and cardiac microvascular function, that is, FRS (including age, active smoking, diabetes, systolic blood pressure, total cholesterol, and high-density lipoprotein), kidney function (measured as creatinine), positive CACS, and use of lipid-lowering medication and/or antihypertensive medication. Test for interaction was performed with a general linear model. With a total of 94 participants in the 2 groups, we obtained a power of 0.8 to detect a difference of 0.4 in MFR (alpha, 0.05) using previously published standard deviations from MPI data using ^82^Rb and adenosine on the same scanner [24].

All statistics were performed using SPSS 22 (IBM SPSS Statistics for Windows, version 22.0; IBM Corp., Armonk, NY).

## RESULTS

The characteristics of the 2 groups are shown in Table 1. From this table, some differences should be noted; for example, the women were almost 10 years younger, were less likely to be on lipid-lowering or antihypertensive treatment, and had a more “favorable” lipid profile and lower creatinine. Also, the FRS was significantly lower among women. However, men and women did not differ significantly regarding active smoking, and their body mass index (BMI) was very similar (borderline overweight). Ninety-eight percent of the men included were white, whereas race was more diverse in the female group. Ninety-six and 98% of the men and women, respectively, were CMV seropositive, all with avidity >80%, and their CMV IgG concentrations did not differ significantly.

**Table 1.** Baseline Characteristics

Parameter	Men	Women	*P* Value
No.	50	44	
Age, mean ± SEM, y	53 ± 1	45 ± 1	<.001
Race, No. (%)			<.001
White	49 (98)	21 (48)	
Black	0	13 (29)	
Asian	0	9 (21)	
Other	1 (2)	1 (2)	
Active smoking, No. (%)	12 (24)	6 (14)	.16
BMI, mean ± SEM, kg/m^2^	24.9 ± 0.5	24.6 ± 0.8	.74
Medication, No. (%)			
Antihypertensive	18 (36)	8 (18)	.04
Statin	13 (26)	3 (7)	.01
Antidiabetics	1 (2)	2 (5)	.45
Clinical CVD, No. (%)^a^	2 (4)	0 (0)	.28
FRS, CHD 10 y, mean ± SEM, %	10.3 ± 0.8	2.7 ± 0.4	<.001
Lipids, mean ± SEM, mmol/L			
Total cholesterol	5.7 ± 0.1	5.4 ± 0.2	.21
HDL	1.4 ± 0.1	1.7 ± 0.1	.02
LDL	3.4 ± 0.1	3.1 ± 0.1	.09
Blood pressure, mean ± SEM, mmHg			
Systolic	127 ± 2	124 ± 3	.51
Diastolic	70 ± 1	80 ± 2	<.001
Creatinine, mean ± SEM, μmol/L	80 ± 2	63 ± 2	<.001
Hepatitis, No. (%)^b^			
Chronic hepatitis B	3 (6)	2 (5)	.56
Chronic hepatitis C	1 (2)	3 (7)	.26
Cytomegalovirus, No. (%)			
CMV positive	48 (96)	43 (98)	.55
CMV IgG, median (range), U/mL^c^	123 (25–924)	89 (34–372)	.22

Abbreviations: BMI, body mass index; CHD, coronary heart disease; CVD, cardiovascular disease; FRS, Framingham risk score; HDL, high-density lipoprotein; LDL, low-density lipoprotein; SEM, standard error of the mean.

^a^Defined as history of myocardial infarction, transient ischaemic attack/stroke, angina, peripheral arterial disease, or revascularization procedure.

^b^Defined as HBsAg positive and hepatitis C IgG positive.

^c^CMV positive only.

### HIV Parameters

All participants included in the study received ART, ~95% had CD4 cell counts >350 10^6^/L, and >90% had viral loads <20 copies/mL. The ART regimens were very similar among men and women, except that women were more likely to receive integrase inhibitors (Table 2).

**Table 2.** HIV-Related Characteristics

Parameter	Men	Women	*P* Value
CD4 cell count, median (range), 10^6^/L	645 (285–1390)	644 (222–1780)	.45
CD4 cell count >350 10^6^/L, No. (%)	47 (94)	42 (95)	.60
Nadir CD4 cell count, median (range), 10^6^/L	157 (0–606)	156 (3–553)	.78
HIV RNA, median (range), copies/mL	19 (19–39)	19 (19–36)	.71
HIV RNA ≤20 copies/mL, No. (%)	47 (94)	41 (93)	.59
HIV duration, mean ± SEM, y	16.5 ± 1	14.4 ± 1	.13
ART duration, mean ± SEM, y	12.6 ± 0.6	11.2 ± 0.7	.13
ART regimens, No. (%)			
≥2 NRTIs + ≥1 NNRTI	31 (62)	24 (55)	.31
≥2 NRTIs + PI	13 (26)	11 (25)	.55
PI only	3 (6)	1 (2)	.36
INI	2 (4)	6 (14)	.09
Other	1 (2)	2 (5)	.54

Abbreviations: ART, antiretroviral treatment; INI, integrase inhibitor; NNRTI, non-nucleoside reverse transcriptase inhibitor; NRTI, nucleoside reverse transcriptase inhibitor; PI, protease inhibitor; SEM, standard error of the mean.

### Myocardial Blood Flow and Myocardial Flow Reserve

**Figure 1.**
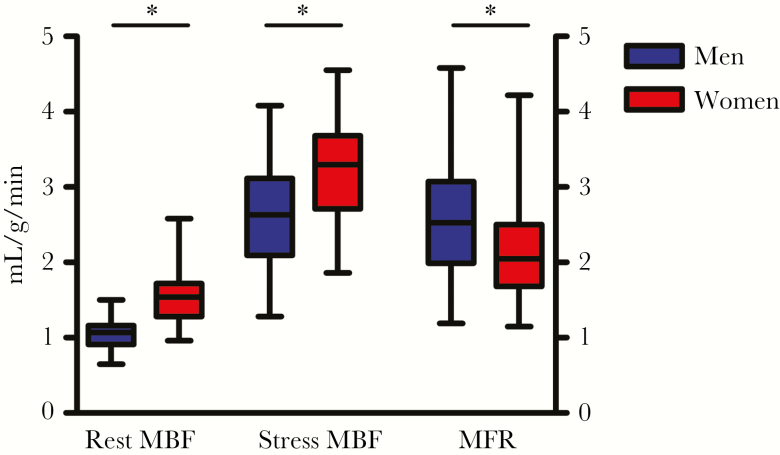
Levels of myocardial blood flow (MBF) at rest and stress in mL/g/min and the unit less myocardial flow reserve (MFR; right y-axis) in men and women living with HIV. ^*^P < .05.

The women had significantly higher MBF at both stress and rest, and when corrected for baseline cardiac work. However, their mean MFR was significantly lower than that of men (2.13 ± 0.10 vs 2.57 ± 0.11; *P =* .003). Significantly more women had an MFR <2 (45% vs 24%; *P =* .03) (Table 3 and Figure 1). Race did not influence the MFR (*P =* .92), nor was an association found between age and MFR (*β* = –.003, *P* = .25 for men; *β* = –.002, *P* = .45 for women).

**Table 3.** Data From the ^82^-Rubidium PET/CT Scan

Parameter	Men (n = 50)	Women (n = 44)	*P* Value
Positive CACS, No. (%)	20 (40)	7 (16)	.01
CACS, median (range)^a^	123 (1–1884)	130 (22–599)	.74
Rest Rb-PET			
Rate-pressure product, mean ± SEM	8742 ± 242	8030 ± 400	.12
LVEF, mean ± SEM, %	54 ± 1	66 ± 1	<.001
Stress Rb-PET			
Rate-pressure product, mean ± SEM	10 558 ± 403	10 231 ± 504	.61
LVEF, mean ± SEM, %	60 ± 1	71 ± 1	<.001
LVEF increase, mean ± SEM, %	13 ± 1	9 ± 1	.04
Perfusion data			
MBF rest, mean ± SEM, mL/g/min	0.89 ± 0.03	1.23 ± 0.05	<.001
MBF rest RPP corrected, mean ± SEM, mL/g/min	1.03 ± 0.03	1.56 ± 0.05	<.001
MBF stress, mean ± SEM, mL/g/min	2.61 ± 0.09	3.2 ± 0.10	<.001
MFR, mean ± SEM	2.57 ± 0.11 (n = 46)	2.13 ± 0.10 (n = 42)	.003
MFR <2, No. (%)	11 (24)	19 (45)	.03
SSS >4, No. (%)	11 (22)	6 (14)	.23

Abbreviations: CACS, coronary artery calcium score; LVEF, left ventricular ejection fraction; MBF, myocardial blood flow; MFR, myocardial flow reserve; PET/CT, positron emission tomography/computed tomography; SEM, standard error of the mean; SSS, summed stress score.

^**a**^For the CACS positive only.

HIV-specific variables had no impact on the MFR: CD4 nadir (*β* = –.03, *P* = .55 for men; *β* = .004, *P* = .93 for women), current CD 4 cell count (*β* = –.05, *P* = .67 for men; *β* = –.03, *P* = .80 for women), duration of ART (*β* = .002, *P* = .60 for men; *β* = 0.001, *P* = .90 for women). No significant interaction between sex and the covariates was found.

Twenty-two percent of the men and 14% of the women had an SSS >4 (*P* = .23), and in sensitivity analyses that excluded subjects with an SSS >4, reflecting obstructive coronary artery disease, the mean MFR remained lower among women (2.17 ± 0.11 vs 2.61 ± 0.13; *P* = .014).

### Cytomegalovirus IgG and MFR

**Figure 2.**
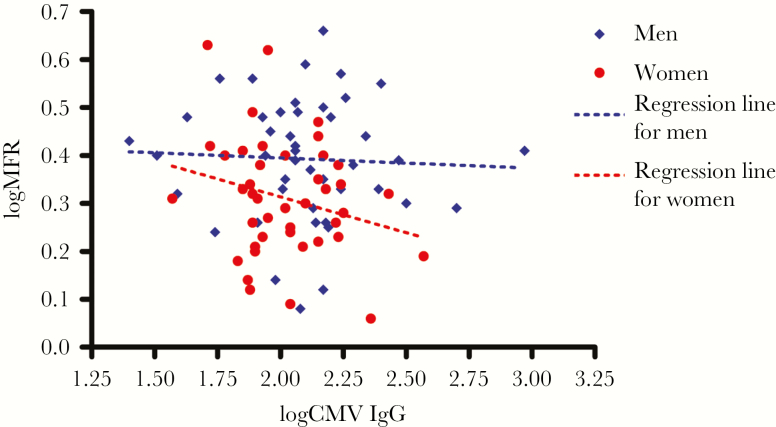
Scatter plots with regression lines of cytomegalovirus (CMV) immunoglobulin G (IgG) vs myocardial flow reserve (MFR).

A significant negative association was found between CMV IgG and MBF at stress among the women (*β* = –.20, *P* = .003) but not men (*β* = .03, *P* = .59, *P* for interaction = .01), whereas no association was found for CMV IgG and MBF at rest. In the group of women, a trend for decrease in MFR with an increase in CMV IgG was found; however, it was not significant (*β* = –.15, *P* = .12). No association was found among men (*β* = –.02, *P* = .77) (Figure 2).

In a multiple regression analysis adjusting for possible confounders of MFR, we found a significant negative association of CMV IgG and MFR among women (*β* = –.33, *P* = .004) but not men (*β* = .03, *P* = .73). In this analysis, a borderline significant interaction between sex and CMV IgG was found (*P* for interaction = .05). CMV IgG was not associated with risk of having a positive CACS or SSS >4 in either group (odds ratio [OR] per log_10_ increase in CMV IgG for women: OR, 0.1–25 for positive CACS and 0.1–27 for SSS >4; men: OR, 0.6–85 for positive CACS and 0.4–40 for SSS >4).

## DISCUSSION

In this first study to compare cardiac microvascular function between women and men living with HIV, we found that the MFR in WLWH with viral suppression, normal CD4 cell counts, and very low cardiovascular risk scores was significantly lower than in MLWH. Forty-five percent of the women included in this study had an MFR <2, indicating that the increased risk of CVD among WLWH could be explained by a compromise in cardiac microvascular function. Further, the decrease in microvascular function appeared to be associated with CMV IgG.

In the general population, MFR has a high predictive power for future cardiovascular events [25–27], and it seems that the MFR is a more reliable measure than MBF alone in both risk prediction and reclassification ability [25, 26, 28]. Importantly, the prognostic ability of MFR is unaffected by sex [13, 14]. In a large study of 1218 patients including 813 women with no signs of obstructive coronary artery disease on semiquantitative visual analysis followed for a median of 1.3 years, MACE occurred earlier and more frequently among both men and women with CMD, defined as MFR <2.0, than in those without CMD regardless of sex [13].

We found that the women in our study had a mean MFR comparable to that of a cohort of 107 women with angina from the iPOWER study who underwent ^82^Rb PET/CT on the same scanner with identical acquisition, reconstruction protocols, and cardiac image analysis software during the same period [29]. The mean age in that study was 61.8 years, and cardiovascular risk factors were more prevalent. These patients had a median MFR (interquartile range) of 2.13 (1.80–2.40), very similar to the WLWH in the present study who had a mean MFR of 2.13 despite being more than 15 years younger and having no apparent risk of CVD.

Few studies have looked at cardiac vasomotor function in PLWH. Two previous studies found that MLWH with low CVD risk have MFRs comparable to HIV-uninfected men [16, 30]. On the other hand, a recent study of PLWH both with and without CAD found that PLWH (63% men) *without* CAD had coronary endothelial function, as assessed by coronary MRI, comparable to HIV-uninfected patients *with* CAD, with no obvious influence of sex [31]. These studies used different imaging modalities, including cardiac stress testing protocols, which arguably could lead to different results. Studies looking specifically at subclinical CVD in WLWH have found a higher prevalence of carotid lesions than in HIV-uninfected women but comparable to MLWH [32]. However, a recent study of subclinical coronary lesions in PLWH on ART found that the prevalence of any type of coronary lesion (including high-risk plaques) among WLWH was significantly lower than among MLWH. The authors suggest that the increased risk of CVD among WLWH despite lower prevalence of coronary lesions could be explained by microvascular disease [33]. Our study supports this theory and extends the findings by suggesting a pathophysiologic role of CMV. In the general population, CMV has been associated with endothelial dysfunction [34] and detected within atherosclerotic plaques [35], and a recent meta-analysis found that CMV infection is associated with an increased risk of CVD [36]. Among PLWH, co-infection with CMV is higher than in the general population [8] and seems to cause an immune response involved in the pathogenesis of CVD [8, 37].

However, in this study, we only found an association between MFR and CMV among women. Therefore, questions arise pertaining to the difference in sex. Do women react differently to CMV IgG regarding vascular pathology, or is the CMV IgG correlated with more inflammation in WLWH? Indeed, some studies suggest that inflammation and immune dysfunction may be more prevalent in WLWH compared with MLWH [38, 39].

Our study is limited by the inability to investigate soluble markers of inflammation, and further studies are therefore warranted to explore how chronic inflammation influences cardiac microvascular function in PLWH. Also, the quantification of CMV IgG only reflects 1 aspect of co-infection with CMV, which is a highly immunogenic virus with impact on both the cellular and adaptive immune systems. Underlying immunological and inflammatory perturbations may therefore have gone unaccounted for in this study.

Finally, we did not have data on menopause, nor did we have the possibility to measure endogenous sex hormone production. This could be of importance as endogenous estrogen production may protect women against CVD [40] and perturbation of sex hormone levels has been proposed to impact the risk of CVD among WLWH [41].

On the other hand, age was not associated with MFR in this study, and when comparing a premenopausal and (peri)menopausal group dichotomized at 47 years [42], we found no difference in MFR. This indicates that cardiac microvascular dysfunction seems to affect WLWH at all ages. In this cross-sectional study, we were unable to determine if the low MFR found among the women will translate into clinical CVD and/or MACE.

## CONCLUSION

In this first study comparing MFR using ^82^Rb PET MPI in women and men living with HIV on stable ART, we found that WLWH had significantly lower MFR than MLWH who were older and had more cardiovascular risk factors. The MFR found among WLWH was comparable to that of a previously published cohort of HIV-uninfected women with angina, who were more than 15 years older and had higher CVD risk. Further, our study indicates an association between CMV infection and the observed sex difference in MFR. These findings call for further studies into both the prevalence and pathogenesis of cardiac microvascular dysfunction in WLWH, especially as traditional cardiovascular screening tools do not readily capture this pathology. Finally, this study underlines the need for continued awareness of the increased cardiovascular risk found among WLWH of young age and low predicted risk scores.
